# Transcranial Direct Current Stimulation for Anxiety During Laparoscopic Colorectal Cancer Surgery: A Randomized Clinical Trial

**DOI:** 10.1001/jamanetworkopen.2024.6589

**Published:** 2024-04-18

**Authors:** Chunyan Li, Mingshu Tao, Dexian Chen, Qi Wei, Xingyu Xiong, Wenxin Zhao, Wen Tan, Jie Yang, Yuan Han, Hongxing Zhang, Song Zhang, He Liu, Jun-Li Cao

**Affiliations:** 1 Department of Anesthesiology, the Affiliated Hospital of Xuzhou Medical University, Xuzhou, China; 2 National Medical Products Administration Key Laboratory for Research and Evaluation of Narcotic and Psychotropic Drugs, Xuzhou Medical University, Xuzhou, China; 3 Jiangsu Province Key Laboratory of Anesthesiology, Xuzhou Medical University, Xuzhou, China; 4 Jiangsu Key Laboratory of Applied Technology of Anesthesia and Analgesia, Xuzhou Medical University, Xuzhou, China; 5 Department of Anesthesiology, Eye & ENT Hospital of Fudan University, Shanghai, China; 6 Department of Anesthesiology, Renji Hospital and Shanghai Jiaotong University School of Medicine, Shanghai, China; 7 Department of Anesthesiology & Clinical Research Center for Anesthesia and Perioperative Medicine, Huzhou Central Hospital, Huzhou, China; 8 Department of Anesthesiology & Clinical Research Center for Anesthesia and Perioperative Medicine, The Fifth School of Clinical Medicine of Zhejiang Chinese Medical University, Huzhou, China; 9 Department of Anesthesiology & Clinical Research Center for Anesthesia and Perioperative Medicine, The Affiliated Huzhou Hospital, Zhejiang University School of Medicine, Huzhou, China; 10 Department of Anesthesiology & Clinical Research Center for Anesthesia and Perioperative Medicine, The Affiliated Central Hospital, Huzhou University School of Medicine, Huzhou, China; 11 Huzhou Key Laboratory of Basic Research and Clinical Translation for Neuromodulation, The Fifth School of Clinical Medicine of Zhejiang Chinese Medical University, Huzhou, China

## Abstract

**Importance:**

Perioperative anxiety is prevalent among patients undergoing surgical treatment of cancer and often influences their prognosis. Transcranial direct current stimulation (tDCS) has shown potential in the treatment of various anxiety-related disorders, but data on the impact of tDCS on perioperative anxiety are limited.

**Objective:**

To evaluate the effect of tDCS in reducing perioperative anxiety among patients undergoing laparoscopic colorectal cancer (CRC) resection.

**Design, Setting, And Participants:**

This randomized clinical trial was conducted from March to August 2023 at the Affiliated Hospital of Xuzhou Medical University. Patients aged 18 years or older undergoing elective laparoscopic radical resection for CRC were randomly assigned to either the active tDCS group or the sham tDCS group. Intention-to-treat data analysis was performed in September 2023.

**Interventions:**

Patients were randomly assigned to receive 2 sessions of either active tDCS or sham tDCS over the left dorsolateral prefrontal cortex on the afternoon of the day before the operation and in the morning of the day of operation.

**Main Outcomes and Measures:**

The main outcome was the incidence of perioperative anxiety from the day of the operation up to 3 days after the procedure, as measured using the Hospital Anxiety and Depression Scale-Anxiety (HADS-A) subscale (range: 0-21, with higher scores indicating more anxiety). Secondary outcomes included postoperative delirium (assessed by the Confusion Assessment Method or Confusion Assessment Method intensive care unit scale); pain (assessed by the 10-point Numeric Rating Scale [NRS], with scores ranging from 0 [no pain] to 10 [worst pain]); frailty (assessed by the Fatigue, Resistance, Ambulation, Illness and Loss of Weight [FRAIL] Index, with scores ranging from 0 [most robust] to 5 [most frail]; and sleep quality (assessed by the Pittsburgh Sleep Quality Index [PSQI], with scores ranging from 0 to 21 and higher scores indicating worse sleep quality) after the 2 sessions of the tDCS intervention.

**Results:**

A total of 196 patients (mean [SD] age, 63.5 [11.0] years; 124 [63.3%] men) were recruited and randomly assigned to the active tDCS group (98 patients) or the sham tDCS group (98 patients). After the second tDCS intervention on the day of the operation, the incidence of perioperative anxiety was 38.8% in the active tDCS group and 70.4% in the sham tDCS group (relative risk, 0.55 [95% CI, 0.42-0.73]; *P* < .001). Patients in the active tDCS group vs the sham tDCS group were less likely to have postoperative delirium (8.2% vs 25.5%) and, at 3 days after the operation, had lower median (IQR) pain scores (NRS, 1.0 [1.0-1.0] vs 2.0 [2.0-2.0]), better median (IQR) sleep quality scores (PSQI, 10.5 [10.0-11.0] vs 12.0 [11.0-13.0]), and lower median (IQR) FRAIL Index (2.0 [1.0-2.0] vs 2.0 [2.0-3.0]).

**Conclusions and Relevance:**

Findings of this randomized clinical trial indicate that administration of 2 preoperative sessions of tDCS was associated with a decreased incidence of perioperative anxiety in patients undergoing elective CRC resection. Active tDCS was also associated with better anxiety scores, pain levels, and sleep quality as well as reduced postoperative delirium and frailty. The findings suggest that tDCS may be a novel strategy for improving perioperative anxiety in patients undergoing CRC resection.

**Trial Registration:**

Chinese Clinical Trial Register Identifier: ChiCTR2300068859

## Key Points

**Question:**

What is the effect of transcranial direct current stimulation (tDCS) on the perioperative anxiety of patients undergoing colorectal cancer surgical resection?

**Findings:**

This randomized clinical trial of 196 patients undergoing laparoscopic colorectal cancer resection found a lower incidence of perioperative anxiety among patients who received 2 active tDCS interventions than among patients in the sham tDCS group (39% vs 70%, respectively).

**Meaning:**

The findings of this study suggest that tDCS may be a novel strategy for improving perioperative anxiety in surgical patients and may expand the application of neuromodulatory approaches in the perioperative period.

## Teaser

This randomized clinical trial examines the effect of transcranial direct current stimulation (tDCS) on perioperative anxiety in patients undergoing colorectal cancer (CRC) resection in China.

## Introduction

Colorectal cancer (CRC) is the third most commonly diagnosed malignant neoplasm and the second leading cause of cancer-related deaths worldwide.^1^ As early detection and survival rates of CRC improve, there is increasing concern about the quality of life and psychological health of patients after treatment.^2^

Surgical resection is the primary treatment for CRC.^3^ However, a large observational study showed that surgical treatment is the major cause of perioperative anxiety, which may be one of the most common perioperative psychiatric symptoms experienced by patients with cancer.^4^ The incidence of perioperative patient anxiety ranges from 11% to 80% in different disorders.^5^ In addition, perioperative anxiety and surgical procedures both can lead to a stress response,^6^ and stress hormones are associated with perioperative antimetastatic immunosuppression and tumor-promoting alterations in the microenvironment.^7,8^ Perioperative anxiety is also commonly associated with perioperative neurocognitive disorder, pain, and sleep disturbances, leading to decreased quality of life and increased disease-related morbidity and mortality.^9^ Therefore, early intervention for preoperative anxiety is crucial.

Currently, pharmacologic and nonpharmacologic approaches are commonly used clinically to alleviate perioperative anxiety.^10^ Nevertheless, preanesthetic anxiolytic drugs, such as benzodiazepines and opioids, are not strongly recommended due to their adverse effects, which may lead to respiratory depression and prolonged recovery time.^11^ Nonpharmacological interventions, such as preoperative visits and music therapy, are becoming more popular, and noninvasive central neuromodulation techniques have shown potential to alleviate patients’ preoperative anxiety.^12^

Transcranial direct current stimulation (tDCS) is a noninvasive brain stimulation modality that alters cortical excitability via weak, direct currents between 2 electrodes placed on the scalp.^13^ Currently, tDCS is widely used in the treatment of neurological and psychiatric disorders,^14,15^ and research has focused on the mood and physical effects of tDCS over the dorsolateral prefrontal cortex (DLPFC).^16^ The application of tDCS has shown potential efficacy in the treatment of various anxiety-related disorders, including generalized anxiety disorders^17^ and social anxiety disorders.^18^ Additionally, tDCS has been shown to be beneficial in protecting perioperative brain function and improving peripartum mental health disorders.^19,20^ However, available data on the effect of tDCS on perioperative anxiety are limited.

The use of tDCS is beneficial in the psychoneurological field to alleviate anxiety symptoms and has the advantages of being safe, efficient, portable, and noninvasive. Based on these findings, the primary objective of this study was to assess the effect of tDCS on perioperative anxiety in patients undergoing radical resection for CRC and to provide clinical evidence for tDCS intervention in the alleviation of perioperative anxiety.

## Methods

### Study Design and Population

This prospective, single-center, randomized, double-blind, placebo-controlled clinical trial was conducted at the departments of anesthesiology and gastrointestinal surgery at the Affiliated Hospital of Xuzhou Medical University from March to August 2023. The research protocol was approved by the Ethics Committee of the Affiliated Hospital of Xuzhou Medical University and written informed consent was obtained from all participants before enrollment in this trial. This study followed the Consolidated Standards of Reporting Trials (CONSORT) reporting guideline for randomized clinical trials. The trial protocol and statistical plan are available in Supplement 1.

### Participants

Patients aged 18 years or older who were scheduled for elective laparoscopic radical resection of CRC with American Society of Anesthesiologists physical status classification of III or greater were eligible. Patients were excluded if they refused to sign the consent form, had neuropsychiatric disorders or a previous history of neurological or psychiatric disorders, craniocerebral or scalp injuries, severe cardiovascular and cerebrovascular diseases, metal implants in the body, a Mini-Mental State Examination score (range: 0-30, with lower scores indicating worse cognitive function) lower than 15, or long-term use of psychotropic drugs, such as cortisol or antidepressant or anxiety drugs.

### Randomization and Blinding

Based on a computer-generated random number table, participants were centrally randomized 1:1 to either the active tDCS group or the sham tDCS group. The allocation information was concealed in opaque envelopes and not revealed until the investigators administered the intervention on the day before the operation. Outcome assessors, the researchers who processed data, other health care personnel, and patients were blinded to the treatment allocation.

### Study Procedures and Interventions

On the day before the operation, potential participants were identified from the elective surgery list by a member of the research team. Active tDCS or sham tDCS was administered to the patients in the treatment room of the wards on the afternoon of the day before the surgical procedure and in the morning of the day of the procedure. Electrostimulation was delivered through 2 electrodes placed in saline-soaked sponges. The electrodes were fixed by a stretchy hat, with the anode over the left DLPFC and the cathode over the right DLPFC. The apparatus used in this trial (model MBM-I; Jiangxi Huaheng Jingxing Medical Technology Co) is shown in eFigure 1 in Supplement 2. Patients in the active tDCS group received 2 mA tDCS for 20 minutes with a 30-second ramp-up phase of current at the beginning and a 30-second ramp-down phase at the end. Patients in the sham tDCS group received only a 30-second ramp-up phase of current at the beginning and a 30-second ramp-down phase at the end of each session without a constant current of 2 mA for 20 minutes. The researchers who implemented the intervention (D.C. and W.Z.) closely observed as the patients received the stimulation. Per protocol, if the patient complained of any unbearable discomfort, the stimulation was to be terminated immediately; this did not occur.

Standard monitoring procedures were initiated as soon as patients arrived in the operating room, including electrocardiography, invasive arterial blood pressure monitoring, pulse oximetry, and central venous catheterization. Patients were administered general anesthesia with 0.5 μg/kg of sufentanil, 0.3 mg/kg of etomidate, and 1 mg/kg of rocuronium. The tracheal catheter was inserted after the bispectral index value decreased to less than 60, and the end-expiratory carbon dioxide partial pressure was maintained between 35 and 45 mm Hg by using anesthesia machines for mechanical ventilation. Patients underwent ultrasonography-guided bilateral transversus abdominis plane block with 40 mL of 0.375% ropivacaine. Intravenous infusion of propofol at a rate of 4 to 6 mg/kg/h and remifentanil at a rate of 0.1 to 0.3 μg/kg/min, along with continuous inhalation of 1% sevoflurane, was used to maintain bispectral index values between 40 and 60 for anesthesia maintenance. If required, vasoactive drugs were administered to maintain hemodynamic stability during anesthesia. Patients were transferred to the postanesthesia care unit after the operation. The tracheal catheter was removed after the patient regained consciousness from anesthesia, ensuring an optimal tidal volume. Postoperative analgesia was achieved with a patient-controlled intravenous infusion of 1.5 μg/kg of sufentanil and 6 mg of tropisetron with saline up to 100 mL; the background infusion rate was 2 mL/h and the self-controlled analgesic dose was 0.5 mL.

### Clinical Outcomes and Assessments

The primary outcome was the incidence of perioperative anxiety in patients from the day of the operation to 3 days after procedure after 2 sessions of tDCS intervention. Perioperative anxiety was measured using the Hospital Anxiety and Depression Scale-Anxiety (HADS-A) subscale, a standardized self-report instrument consisting of 7 items. The HADS-A is a commonly used self-rating scale developed to assess psychological distress in nonpsychiatric patients,^21^ and has good sensitivity and specificity.^22^ Patients with a HADS-A score of 8 or more were considered to be experiencing anxiety, with a score of 8 to 10 indicating mild anxiety, 11 to 14 indicating moderate anxiety, and 15 to 21 indicating severe anxiety. In this study, anxiety was defined as occurring if the HADS-A score was 8 or more at any assessment time after 2 sessions of tDCS intervention. On the day before the operation, the researcher assessed the patient for anxiety in the ward treatment room immediately before the first tDCS or sham intervention (T0), followed by a second assessment of anxiety after the first tDCS or sham intervention (T1). On the day of the operation, the researcher conducted separate assessments before (T2) and after (T3) administering the second tDCS or sham stimulus. Anxiety scores were assessed at 2 hours after the surgical procedure (T4), on the first day after procedure (T5), the second day after the procedure (T6), the third day after the procedure (T7), and the third month after the operation (T8).

Secondary outcomes included anxiety scores (T1-T8); the incidence of postoperative delirium (assessed by the Confusion Assessment Method or Confusion Assessment Method intensive care unit scale^23^ at T4-T7); pain scores (assessed by the 10-point Numeric Rating Scale [NRS]^24^ at T4-T7, ranging from 0 [no pain] to 10 [worst pain]); frailty scores (assessed by the Fatigue, Resistance, Ambulation, Illness and Loss of Weight [FRAIL] Index^25^ at T5-T8; scores range from 0 [most robust] to 5 [most frail]); and sleep quality scores (assessed by the Pittsburgh Sleep Quality Index [PSQI]^26^ at T4-T8; scores range from 0 to 21, with higher scores indicating worse sleep quality). The timeline of this trial is shown in eFigure 1 in Supplement 2.

### Statistical Analysis

The preliminary data showed that the incidence of perioperative anxiety in this patient population was 51.4%, and we assumed that a 40% reduction in the incidence of perioperative anxiety after 2 sessions of tDCS intervention would be considered effective for its treatment. Using a study power of 0.80 and an α = .05, we then calculated that 88 patients per group were required by using PASS, version 15.0 (NCSS). Assuming a 10% loss to follow-up, we aimed to recruit 98 participants in each group.

Data analysis was performed in September 2023. Since missing data were about 1% for the primary outcome and less than 10% for all secondary outcomes, no imputation was conducted on missing data. All analyses were conducted according to the intention-to-treat principle.

The primary outcome, the incidence of perioperative anxiety in patients from the day of the operation to 3 days after the operation after 2 sessions of active or sham tDCS intervention, was compared using the χ^2^ test or Fisher exact test, with differences between groups expressed as relative risk (RR) and 95% CIs. Because the sample size target was based on preliminary data, we used PASS, version 15.0 to confirm that the incidence of perioperative anxiety in this trial was sufficient to test efficacy. The linear mixed-effects model was used to compare the secondary outcomes, with baseline (T0) values as covariates; treatment, time, and the interaction of treatment-by-time as fixed effects (tested by analysis of variance); and the patient’s random intercept as a random effect to account for differences in time. The treatment-by-time interaction term was tested first. If significant, between- and within-treatment differences were tested at each time point, and analyses were adjusted for multiple comparisons using the Bonferroni test for differences in within-treatment anxiety scores from baseline (T0). Otherwise, the main effect of treatment was tested next, and no Bonferroni correction was made for assessing the treatment effect at each time point. The Kolmogorov-Smirnov test was used to assess normality. The independent sample *t* test was used for normally distributed data, and the Mann-Whitney *U* test was used otherwise for nonnormally distributed data. Continuous variables with normal distribution were represented by means and SDs, and variables with nonnormal distribution were represented by medians and IQRs. Categorical variables were expressed as frequencies and proportions and analyzed using the χ^2^ test or Fisher exact test. The RRs and 95% CIs were used to describe the differences in dichotomous outcomes.

In the exploratory analysis, a post hoc sensitivity analysis excluding the preoperative nonanxious or only mildly anxious population from baseline (T0) was conducted, using a linear mixed-effects model to explore the impact on the overall study conclusions. Additionally, the influence of different variables on postoperative anxiety (T4) was assessed in logistic regression models. Patients were divided into anxious and nonanxious groups based on anxiety scores at T4. Variables with *P* < .10 in univariable analyses were included in the multivariable logistic regression model to control for confounding factors. We used variance inflation factors to examine possible collinearity among covariates, and group was added to the multivariable model as a variable of interest. We checked the model fitness for the logistic regression using the Hosmer-Lemeshow goodness-of-fit test statistics. Finally, another post hoc analysis was conducted to determine the heterogeneity of tDCS in the subgroup of patients with postoperative anxiety at T4, and the effect of the intervention method is presented separately by each subgroup (sex, PSQI score, ileostomy status, adverse event, and need for remedial analgesia). The interaction between these variables and treatment was determined by binary logistic regression. Data were analyzed using SPSS, version 25.0 (IBM Corp) and hypothesis tests were 2-sided with significance set at *P* < .05.

## Results

### Study Population

A total of 271 patients scheduled for elective laparoscopic radical resection of CRC were screened for eligibility from March to August 2023. In all, 44 patients declined to participate in the trial, 23 patients did not meet inclusion criteria (12 patients because of severe cardiovascular and cerebrovascular diseases, 3 patients because of a cardiac pacemaker, and 8 patients because of previously diagnosed anxiety disorder or long-term oral benzodiazepine use), and 8 patients cancelled the operation. Ultimately, 196 patients (mean [SD] age, 63.5 [11.0] years; 124 men [63.3%] and 72 women [36.7%]) were enrolled and randomly assigned to either the active tDCS group (n = 98) or the sham tDCS group (n = 98) and completed the trial. In the sham tDCS group, 5 patients were lost to follow-up (1 patient was admitted to the intensive care unit due to hypoxia, 1 patient underwent a surgical procedure for postoperative intracranial hemorrhage, and 3 patients died within 3 months after the operation) and in the active tDCS group, 2 patients were lost to follow-up (2 patients died within 3 months after the operation). The participant flow diagram is shown in the Figure, and the demographic and intraoperative characteristics are detailed in Table 1 and Table 2. In total, 117 patients (59.7%) underwent laparoscopic rectal resection.

**Figure.**
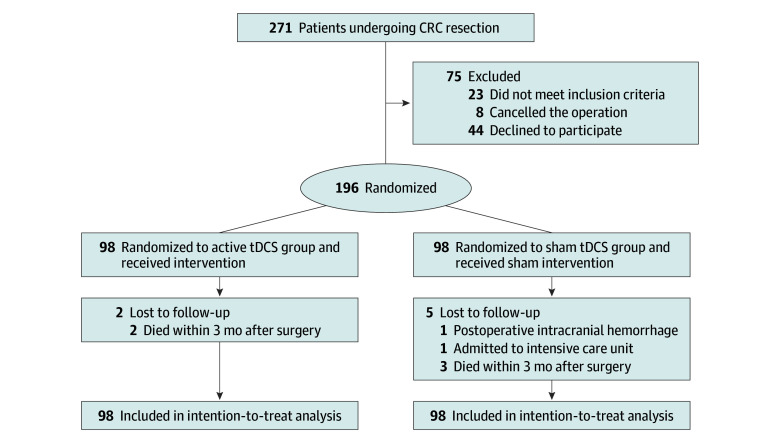
Study Flow Diagram CRC indicates colorectal cancer; tDCS, transcranial direct current stimulation.

**Table 1.** Baseline Characteristics of Study Participants in the Active Transcranial Direct Current Stimulation (tDCS) and Sham tDCS Groups

Characteristic	Participants, No. (%)	
	Active tDCS (n = 98)	Sham tDCS (n = 98)
Age, mean (SD), y	63.5 (10.3)	63.5 (11.8)
Sex		
Male	60 (61.2)	64 (65.3)
Female	38 (38.8)	34 (34.7)
BMI, mean (SD)	24.7 (3.6)	23.4 (3.7)
Education level		
Less than elementary school	11 (11.2)	9 (9.2)
Elementary school	36 (36.7)	39 (39.8)
Middle school	27 (27.6)	30 (30.6)
Technical secondary school	14 (14.3)	10 (10.2)
High school	5 (5.1)	3 (3.1)
College graduate	5 (5.1)	7 (7.1)
ASA classification		
II	89 (90.8)	87 (88.7)
III	9 (9.2)	11 (11.2)
History of surgical procedures	29 (29.6)	37 (37.6)
Comorbidities		
Diabetes	15 (15.3)	11 (11.2)
Hypertension	24 (24.5)	20 (20.4)
Stroke	5 (5.1)	3 (3.1)
Coronary artery disease	2 (2.0)	4 (4.1)
Age-adjusted Charlson Comorbidity Index, median (IQR)	2 (2-3)	2 (1-2)
Mini-Mental State Examination score, median (IQR)	20 (18-21)	20 (18-22)
Hemoglobin level, median (IQR), g/dL	13.00 (11.38-14.00)	12.55 (10.95-13.70)
Numeric Rating Scale score for pain, median (IQR)	0	0
Anxiety	46 (46.9)	47 (48.0)
HADS-A, median (IQR)	7 (7-12)	7 (7-12)
FRAIL scores		
Robust	55 (56.1)	50 (51.0)
Prefrail	40 (40.8)	41 (41.8)
Frail	3 (3.1)	7 (7.1)

Abbreviations: ASA, American Society of Anesthesiologists physical status classification; BMI, body mass index (calculated as weight in kilograms divided by height in meters squared); FRAIL, Fatigue, Resistance, Ambulation, Illness and Loss of Weight; HADS-A, Hospital Anxiety and Depression Scale-Anxiety subscale (range: 0-21, with higher scores indicating greater anxiety).

SI conversion: To convert hemoglobin level to grams per deciliter, multiply by 10.

**Table 2.** Intraoperative and Postoperative Data Between the Active Transcranial Direct Current Stimulation (tDCS) and Sham tDCS Groups

Characteristic	No. (%)		*P* value
	Active tDCS (n = 98)	Sham tDCS (n = 98)	
Duration of operation, median (IQR), min	185.0 (170.0-205.0)	180.0 (160.0-210.0)	.39
Duration of anesthesia, median (IQR), min	222.0 (207.3-241.3)	219.5 (198.8-243.0)	.24
Infusion quantity, median (IQR), mL	1500.0 (1500.0-2000.0)	1500.0 (1500.0-1812.5)	.09
Estimated blood loss, median (IQR), mL	30.0 (25.0-35.0)	30.0 (25.0-35.0)	.60
Transverse abdominis plane block	88 (89.8)	86 (87.8)	.65
Type of operation			
Left hemicolectomy	5 (5.1)	7 (7.1)	.84
Right hemicolectomy	20 (20.4)	18 (18.4)	
Sigmoid resection	16 (16.3)	13 (13.3)	
Rectal resection	57 (58.2)	60 (61.2)	
Ileostomy	35 (35.7)	33 (33.7)	.69
Pathological type			
Adenocarcinoma	92 (93.9)	91 (92.9)	.77
Other^a^	6 (6.1)	7 (7.1)	
Duke stage of CRC			
A	18 (18.4)	20 (20.4)	.96
B	45 (45.9)	47 (48.0)	
C1	27 (27.6)	25 (25.5)	
C2	7 (7.1)	5 (5.1)	
D	1 (1.0)	1 (1.0)	
Maximum tumor diameter, median (IQR), cm	4.3 (3.2-5.5)	5.0 (3.3-6.6)	.17
Tumor metastasis			
Lymph node metastasis	32 (32.7)	26 (26.5)	.63
Distant metastasis	4 (4.1)	4 (4.1)	
ICU admission	2 (2.0)	6 (6.1)	.27
Duration of hospitalization, median (IQR), d	13 (11-16)	14 (11-16)	.75

Abbreviations: CRC, colorectal cancer; ICU, intensive care unit.

^a^ Includes squamous carcinoma, signet ring cell carcinoma, and non-Hodgkin lymphoma.

### Outcomes

After the second tDCS intervention on the day of the operation (T3), the incidence of perioperative anxiety was significantly lower in the active tDCS group compared with the sham tDCS group (38 of 98 patients [38.8%] vs 69 of 98 patients [70.4%]; RR, 0.55 [95% CI, 0.42-0.73]; *P* < .001). There was an overall downward trend in anxiety scores in the active tDCS group throughout the trial, whereas in the sham tDCS group there was a sloping increase in preoperative anxiety scores on the day of the operation and a slow decline after the operation (eFigure 2 in Supplement 2). The mean (SD) HADS-A score decreased from 8.4 (2.7) at T2 (immediately before the second tDCS intervention on the day of the operation) to 6.2 (1.2) at T7 (3 days after the operation) in the active tDCS group, whereas corresponding mean (SD) HADS-A scores in the sham tDCS group decreased from 10.8 (4.0) to 7.5 (1.9) (at T2, *P* < .001; at T7, *P* = .001) (Table 3). Further sensitivity analysis showed that for patients who were moderately to severely anxious at T0, anxiety scores after stimulation were significantly lower in the active tDCS group than in the sham tDCS group; for example, at T3 (immediately after the second tDCS intervention), mean (SD) HADS-A scores were 10.2 (1.9) in the active tDCS group and 15.4 (2.2) in the sham tDCS group (*P* < .001) (eTable 1 in Supplement 2).

**Table 3.** Comparison of Anxiety Between the Active Transcranial Direct Current Stimulation (tDCS) and Sham tDCS Groups

Variable	No. (%)		χ^2^	*df*	*P* value
	Active tDCS (n = 98)	Sham tDCS (n = 98)			
Patients with anxiety					
T0	46 (46.9)	47 (48.0)	0.02	1	.89
T1	46 (46.9)	47 (48.0)	0.02	1	.89
T2	40 (40.8)	68 (69.4)	16.17	1	<.001
T3	38 (38.8)	69 (70.4)	19.78	1	<.001
T4	30 (30.6)	56 (57.1)	14.00	1	<.001
T5	25 (25.5)	51 (52.0)	14.52	1	<.001
T6	20 (20.4)	46 (46.9)	15.44	1	<.001
T7	14 (14.3)	41(41.8)	18.43	1	<.001
T8	11 (11.2)	30 (30.6)	11.13	1	.001
HADS-A score, mean (SD)^a^					
T0	9.5 (3.2)	9.5 (3.5)	NA	NA	.94
T1	8.9 (2.7)^b^	9.5 (3.5)	NA	NA	.13
T2	8.4 (2.7)^b^	10.8 (4.0)	NA	NA	<.001
T3	7.9 (2.3)^b^	10.9 (4.0)	NA	NA	<.001
T4	7.3 (1.9)^b^	9.6 (3.2)	NA	NA	<.001
T5	6.9 (1.7)^b^	8.9 (2.8)	NA	NA	<.001
T6	6.6 (1.5)^b^	8.1 (2.3)^c^	NA	NA	<.001
T7	6.2 (1.2)^b^	7.5 (1.9)^c^	NA	NA	.001
T8	6.1 (1.1)^b^	7.2 (1.7)^c^	NA	NA	.004

Abbreviations: HADS-A, Hospital Anxiety and Depression Scale-Anxiety subscale (range: 0-21, with higher scores indicating greater anxiety); NA, not applicable; T0, before the first tDCS intervention (active or sham) on the day before the operation; T1, after the first tDCS intervention on the day before the operation; T2, before the second tDCS intervention on the day of the operation; T3, after the second tDCS intervention on the day of the operation; T4, 2 hours after the operation; T5, the first day after the operation; T6, the second day after the operation; T7, the third day after the operation; T8, the third month after the operation.

^a^ Anxiety scores were compared in linear mixed-effects models between and within groups (*P* for group <.001, *P* for time <.001, *P* for interaction <.001; analysis of variance *F* for groups = 20.58, *F* for time = 89.12, *F* for interaction = 50.45).

^b^ *P* < .001 for each time point vs T0 within the active tDCS group.

^c^ *P* < .001 for each time point vs T0 within the sham tDCS group.

The incidence of postoperative delirium at any time during the first 3 postoperative days was significantly lower in the active tDCS group (8 of 98 patients [8.2%] vs 25 of 98 patients [25.5%]; RR, 0.32 [95% CI, 0.15-0.67]; *P* = .001) (Table 4). Compared with the sham tDCS group, the active tDCS group had lower NRS pain scores, PSQI scores, and FRAIL scores (Table 4; eFigure 2 in Supplement 2). For example, at T7 (3 days after the operation), the active tDCS group compared with the sham tDCS group had a lower median (IQR) NRS pain score (1.0 [1.0-1.0] vs 2.0 [2.0-2.0]), a lower median (IQR) PSQI score (10.5 [10.0-11.0] vs 12.0 [11.0-13.0]), and a lower median (IQR) FRAIL score (2.0 [1.0-2.0] vs 2.0 [2.0-3.0]) (all *P* < .001).

**Table 4.** Comparison of Other Outcomes Between the Active Transcranial Direct Current Stimulation (tDCS) and Sham tDCS Groups

Variable	No. (%)		*P* value
	Active tDCS (n = 98)	Sham tDCS (n = 98)	
Postoperative delirium	8 (8.2)	25 (25.5)	.001
Type of delirium			
Hypoactive	5 (5.1)	15 (15.3)	.007
Hyperactive	3 (3.1)	7 (7.1)	
Mixed	0	3 (3.1)	
NRS, median (IQR)^a^			
T0	0	0	1.00
T4	3.0 (3.0-3.0)	3.0 (3.0-5.0)	<.001
T5	2.0 (2.0-2.0)	3.0 (2.0-4.0)	<.001
T6	1.5 (1.0-2.0)	2.0 (2.0-3.0)	<.001
T7	1.0 (1.0-1.0)	2.0 (2.0-2.0)	<.001
PSQI, median (IQR)^a^			
T0	12.0 (11.0-14.0)	12.0 (11.0-15.0)	.14
T4	12.0 (12.0-15.0)	15.0 (13.0-16.0)	<.001
T5	12.0 (11.0-13.0)	14.0 (13.0-15.0)	<.001
T6	11.0 (10.0-12.0)	13.0 (12.0-14.0)	<.001
T7	10.5 (10.0-11.0)	12.0 (11.0-13.0)	<.001
T8	10.0 (10.0-11.0)	12.0 (11.0-12.0)	<.001
FRAIL score, median (IQR)^a^			
T0	0.0 (0.0-1.0)	0.0 (0.0-1.0)	.33
T5	3.0 (3.0-3.0)	3.0 (3.0-4.0)	.003
T6	2.0 (2.0-3.0)	3.0 (2.0-3.0)	<.001
T7	2.0 (1.0-2.0)	2.0 (2.0-3.0)	<.001
T8	0.0 (0.0-1.0)	1.0 (1.0-2.0)	<.001

Abbreviations: FRAIL, Fatigue, Resistance, Ambulation, Illness and Loss of Weight (range, 0 [most robust] to 5 [most frail]); NRS, Numeric Rating Scale for pain (range: 0 [no pain] to 10 [worst pain]); PSQI, Pittsburgh Sleep Quality Index (range: 0-21, with higher scores indicating worse sleep quality); T0, before the first tDCS intervention (active or sham) on the day before the operation; T4, 2 hours after the operation; T5, the first day after the operation; T6, the second day after the operation; T7, the third day after the operation; T8, the third month after the operation.

^a^ The NRS, PAQI, and FRAIL scores were compared in linear mixed-effects models between groups (*P* for group <.001, *P* for time <.001, *P* for interaction <.001).

To explore the risk factors for anxiety at 2 hours after the operation (T4), univariable analysis showed that the *P* values of treatment group, preoperative HADS-A score, age, sex, hemoglobin level, ileostomy status, adverse event, remedial analgesia, PSQI score, FRAIL scores, and education level were less than .10; thus, these variables were included in multivariable logistic models. The χ^2^ value of the Hosmer-Lemeshow test was 3.94 (*df* = 8, *P* = .86), and the goodness of fit of this model was outstanding. As shown in eTable 2 in Supplement 2, tDCS intervention, preoperative anxiety level, ileostomy status, and FRAIL scores were independently associated with postoperative anxiety. The interaction term of the subgroup analysis serves as a statistical test to evaluate the impact of the intervention, which is visually presented in the forest plot in eFigure 3 in Supplement 2. The tDCS intervention was associated with a lower incidence of perioperative anxiety in patients with prefrailty or frailty (odds ratio, 0.55 [95% CI, 0.34-0.90]; *P* = .01).

With regard to adverse events, skin tingling occurred at the site of irritation, which patients reported as transient and tolerable. The incidence of skin tingling was higher in the active tDCS group than in the sham tDCS group (11 of 98 patients [11.2%] vs 3 of 98 patients [3.1%]; *P* = .03; eTable 3 in Supplement 2).

## Discussion

This prospective randomized clinical trial found that 2 sessions of anodal tDCS over the left DLPFC, administered the afternoon before the operation and in the morning of the day of operation, was associated with a reduction in the incidence of perioperative anxiety among patients undergoing laparoscopic CRC resection. In addition, this study found that the active tDCS mode may significantly reduce anxiety, alleviate pain, improve sleep quality, as well as decrease the occurrence of postoperative delirium and frailty.

Perioperative anxiety has been recognized as a factor affecting anesthetic management and surgical outcomes,^27^ and is considered the most common psychological stress response in perioperative patients.^4^ In this trial, anxiety has been explained as an emotional state, which is characterized by restlessness, worried thoughts, and physiological arousal.^28^ In this context, tDCS may have a crucial role in alleviating anxiety symptoms by exerting a direct inhibitory influence on the amygdala through modulation of the prefrontal cortex^29^ and exerting an anxiolytic effect by activating cannabinoid receptor 1 in the amygdala.^30^ In line with a previous study,^31^ this trial confirmed that tDCS was effective in reducing the incidence of perioperative anxiety. Notably, a score of 8 or higher on the HADS-A subscale is defined as anxiety, and after 2 sessions of tDCS intervention (T3), the active tDCS group had anxiety scores of less than 8 before the operation, whereas scores in the control group still exceeded 8, which is considered a clinically significant score for reducing perioperative anxiety. In addition, we conducted a post hoc analysis of patients with moderate to severe preoperative anxiety (T0) and found that anxiety scores in the active tDCS group showed an overall decreasing trend and were lower than 8 on the second day after the operation (T6), whereas anxiety scores in the sham tDCS group reached a peak on the day of the operation and remained higher than 8 from the perioperative period to 3 months after the operation.

The prevalence of chronic postoperative anxiety disorders has been reported to range from 11.6% to 22.3%.^32,33^ and cancer survivors have twice the prevalence of postoperative anxiety disorders compared with the general population as a result of postoperative radiation and chemotherapy as well as impaired quality of life after surgical treatment.^34^ As we all know, the presence of mild anxiety or stress may facilitate postoperative recovery by activating the body’s stress response mechanisms,^35,36^ whereas excessive anxiety is often accompanied by autonomic nervous system dysfunction, subjective distress, and impaired social functioning. Unfortunately, an anxiety state lasting 3 months has the potential to develop into a chronic anxiety disorder,^37^ which is why we chose to follow up patients at 3 months postoperatively. We found that tDCS relieved patients’ anxiety 3 months postoperatively, which indicates that preoperative intervention with tDCS may prevent the chronicity of perioperative anxiety. This finding may be related to the long-term effects of tDCS primarily involved in neuronal plasticity.^38,39^

Previous studies have reported that patients with anxiety-related disorders can present an imbalance between the activity of the right and the left DLPFC, with hypoactivity in the left side and hyperactivity in the right side.^40^ In neuroimaging studies, an inverse association has been observed between anxiety and left DLPFC activity.^41^ The modulation of DLPFC activity and plasticity by the tDCS may contribute to its potential therapeutic effects in mood and anxiety disorders.^42^ Therefore, the left DLPFC was chosen as the target for tDCS in this study.

In addition, the application of a single session of tDCS targeting the left DLPFC can promptly alleviate symptoms associated with anxiety states.^28^ Nishida et al^43^ found that the accumulation of immediate neural responses to a single session of tDCS may potentially modulate the stable state of brain activity and ultimately relieve the symptoms of anxiety. When patients with anxiety disorders were treated with more sessions of tDCS, both anxiety and cognitive symptoms improved, with the effect persisting at 2 months of follow-up.^44^ Hence, 2 sessions of tDCS were given prior to the operation to achieve early intervention and anxiety relief through a cumulative effect.

The association between preoperative anxiety and postoperative delirium has been substantiated by research.^45^ It is known that tDCS may also alleviate chronic anxiety disorders, reduce chronic pain, and improve cognitive function by modulating brain-derived neurotrophic factors and decreasing levels of inflammatory factors.^44,46^ The active tDCS group in this trial exhibited a significantly lower incidence of postoperative delirium, which was consistent with findings of our previous study.^19^ In addition, tDCS can enhance the function of the descending pain modulatory system and target corticothalamic pathways of sleep-wake regulation from a top-down perspective.^47,48^ The application of active tDCS in this trial has been advantageous in the reduction of postoperative pain and enhancement of postoperative sleep quality. This study also found that tDCS relieved patients’ postoperative frailty, which may be related to the fact that tDCS can reduce the incidence of postoperative delirium, improve sleep quality, as well as relieve pain.

We are also concerned about the safety of tDCS. A meta-analysis of more than 33 000 sessions of tDCS showed that some patients can experience mild headache or localized tingling, but no long-term adverse effects were reported.^49^ Consistent with findings of the current study, there were no significant adverse treatment-related events. Transient and tolerable skin tingling occurred at the site of irritation in 11.2% of our patients in the sham tDCS group. Two patients in the active tDCS group and 3 patients in the sham tDCS group died within 3 months after the operation, which is considered to be death related to disease progression.

### Limitations

This study has limitations. First, this single-center trial focused on patients scheduled for elective laparoscopic radical resection of CRC, and the generalizability and external validity of the findings were limited. Second, the incidence of perioperative anxiety in this trial was slightly lower than the preliminary data used in the sample size calculation, so we used PASS, version 15.0 to test the incidence of the outcome and then derived a study power of 0.99, which was considered to meet the efficacy of the trial. Third, although we found that early tDCS is effective in alleviating anxiety,^43^ future investigations are recommended that include multiple sessions of tDCS for patients at high risk of perioperative anxiety, potentially enhancing therapeutic efficacy. Fourth, the fear of recurrence is a prevailing concern among all patients with cancer.^34^ During the trial, we did not assess patients’ knowledge of their disease conditions, and thus we do not know how this knowledge may have influenced the results. Fifth, this trial did not use objective indicators, such as imaging techniques (eg, electroencephalography) and biological markers (eg, cortisol concentrations), to explore the effects of tDCS on patients. We believe that it would be beneficial to combine tDCS with objective indicators to investigate the effects of tDCS on functional brain regions,^50^ which may enhance the development of personalized treatment for perioperative anxiety.

## Conclusions

In this randomized clinical trial among patients undergoing laparoscopic radical resection of CRC, we found that 2 sessions of active tDCS intervention before the operation may reduce the incidence of perioperative anxiety. It is necessary to consider the use of neuromodulation approaches as part of an overall strategy to relieve perioperative anxiety in patients undergoing CRC resection.

## Notes

Supplement 1.
Trial Protocol

Supplement 2. **eTable 1.** Comparison of Hospital Anxiety and Depression Scale-Anxiety Subscale (HADS-A) Scores of Patients With Moderate to Severe Anxiety at T0 Between Groups **eTable 2.** Binary Logistic Regression Analysis to Identify Postoperative Anxiety at T4 **eTable 3.** Adverse Events in the Trial **eFigure 1.** Timeline of the Trial and Apparatus Used in This Test **eFigure 2.** Comparison of Mean Scores of Anxiety, Numeric Rating Scale, Pittsburgh Sleep Quality Index (PSQI), and Frailty Index of Patients Between the Two Groups **eFigure 3.** Forest Plot of the Subgroup Analysis

Supplement 3.
Data Sharing Statement
