# Supplementary material for: Transcranial Direct Current Stimulation for Anxiety During Laparoscopic Colorectal Cancer Surgery: A Randomized Clinical Trial
Source: JAMA Netw Open. 2024 Apr 18;7(4):e246589. doi: 10.1001/jamanetworkopen.2024.6589 (PMC12527477; doi:10.1001/jamanetworkopen.2024.6589)
Supplement: Supplement 1. — Trial Protocol [file jamanetwopen-e246589-s001.pdf]

CLINICAL STUDY PROTOCOL

TITLE: Effect of Transcranial Direct Current Stimulation on Perioperative Anxiety in Patients undergoing Laparoscopic Colorectal Tumor Surgery: A Randomized Clinical Trial.

PRINCIPAL INVESTIGATOR: Chunyan Li, MD, He Liu, PhD, Jun-Li Cao, PhD.

PROTOCOL SYNOPSIS

**Title:**	Effect of Transcranial Direct Current Stimulation on Perioperative Anxiety in Patients undergoing Laparoscopic Colorectal Tumor Surgery: A Randomized Clinical Trial
**Study Type:**	A prospective, single-center, randomized, double-blind clinical trial
**Corresponding Author:**	Jun-Li Cao, Department of Anesthesiology, The Affiliated Hospital of Xuzhou Medical University, Xuzhou, China. NMPA Key Laboratory for Research and Evaluation of Narcotic and Psychotropic Drugs & Jiangsu Province Key Laboratory of Anesthesiology & Jiangsu Province Key Laboratory of Anesthesia and Analgesia Application Technology, Xuzhou Medical University, Xuzhou 221004, Jiangsu, China.<br>He Liu, Department of Anesthesiology & Clinical Research Center for Anesthesia and Perioperative Medicine, The Fifth School of Clinical Medicine of Zhejiang Chinese Medical University || Huzhou Central Hospital || The Affiliated Huzhou Hospital, Zhejiang University School of Medicine || Affiliated Central Hospital Huzhou University, Huzhou 313003, Zhejiang, China.

**Study Centers:**	The Affiliated Hospital of Xuzhou Medical University
**Ethics**	Approved by the Medical Ethics Committee of the Affiliated Hospital of Xuzhou Medical University (XYFY2022-KL448-01)
**Trial Registration:**	www.chictr.org.cn Identifiers: ChiCTR2300068859

I. STUDY OBJECTIVES

The experience of perioperative anxiety is prevalent among patients undergoing cancer surgery and often exerts a significant impact on the prognosis of surgical patients. The application of transcranial direct current stimulation (tDCS) has been reported to modulate cortical excitability and has demonstrated promising potential in the treatment of various anxiety-related disorders.

The objective of this study was to evaluate the efficacy of tDCS in reducing perioperative anxiety among patients undergoing laparoscopic colorectal tumor surgery.

II. BACKGROUND

A. Perioperative Anxiety

In this trial, anxiety is defining as an emotion characterized by feelings of tension, worried thoughts, and physical changes like increased blood pressure [1]. According to the Anxiety and Depression Association of America, around 40 million people in the United States have an anxiety disorder. It is the most common group of mental illnesses in the country. However, only 36.9% of people with an anxiety disorder receive treatment. Anxiety disorders are the most common of all mental disorders with 30% prevalence in the population, and they significantly contribute to the economic burden of disease.

Perioperative anxiety, which is one of the main psychological stresses experienced by patients who undergo surgery, can cause activation of the sympathetic nervous system and the hypothalamic–pituitary–adrenal [2].

The incidence of perioperative anxiety ranges from 11% to 80% in different disorders. Perioperative anxiety is associated with perioperative neurocognitive disorder, postoperative pain, and postoperative sleep disturbances, resulting in impaired quality of life and increased disease-related morbidity and mortality in patients [3].

B. Dorsolateral Prefrontal Cortex and Amygdala

The dorsolateral prefrontal cortex (DLPFC) and amygdala play important roles in "emotion dysregulation" which has a profound impact on etiologic research of generalized anxiety disorder (GAD). The DLPFC regulates emotional processing, decision-making, and other high-level cognitive functions through extensive connections with other brain regions, and has been shown to play a key role in the pathophysiology of GAD [4]. Altered activation of the DLPFC in patients with GAD has been associated with emotional dysregulation and attention deficit. Anxiety states associated with acute stress can be considered to result from under-activation of the excitability level of the left DLPFC of the brain, as well as abnormalities in the work of the amygdala of the brain [5]. Additionally, functional abnormalities of the amygdala, known as the most prominent "fear-circuit" structure in the brain that plays a central role in automatic affective processing, have been found in most anxiety disorders [6]. The amygdala has also demonstrated responsibility for facilitating perceptual processing and bottom-up emotional control in individuals with GAD.

C. Transcranial Direct Current Stimulation (tDCS)

The tDCS is a noninvasive brain stimulation modality that modulates cortical excitability through the application of weak, direct currents between two

electrodes placed on the scalp [7]. Studies have shown that tDCS can effectively rebalance the excitability levels between the two hemispheres by anodal stimulation over the left DLPFC and cathodal stimulation over the right DLPFC, thereby leading to the alleviation of anxiety [8].

III. METHODS

This is a prospective, single-center, randomized, double-blind, controlled clinical trial conducted in the department of Anesthesiology at the Affiliated Hospital of Xuzhou Medical University from March to August 2023. The research protocol was approved by the Ethics Committee of the affiliated hospital of Xuzhou Medical University (XYFY2022-KL448-01). The trial was registered at the China Clinical Trial Center (http://www.chictr.org.cn/) with the registration identifier ChiCTR2300068859 on March 1, 2023. This report follows the Consolidated Standards of Reporting Trials (CONSORT) reporting guideline for randomized studies.

A. Recruiting Methods

On the day before the operation, potential participants are identified from the elective surgery list by a member of the research team. Patients aged 18 years or older who are scheduled to receive elective laparoscopic radical surgery for colorectal tumors with American Society of Anesthesiologists (ASA) physical status classification ≤III are screened to assess their study eligibility. Written informed consent is obtained from participants before enrolment in this trial.

B. Inclusion Criteria

Patients aged ≥ 18 years scheduled for elective laparoscopic radical surgery of colorectal tumors with ASA ≤III are eligible.

C. Exclusion Criteria

1. Refuse to sign the test consent;
2. Have neuropsychiatric diseases, and have a history of neurological or psychiatric diseases;
3. There are craniocerebral or head injuries;
4. Serious cardiovascular and cerebrovascular diseases;
5. Those with metal implanted devices in the body;
6. Mini-Mental State Examination score less than 15;
7. Long-term use of psychotropic drugs such as cortisol or antidepressants and anxiety.

D. Consent Procedure

All potential subjects who meet the inclusion criteria that are identified by the chief anesthetist and/or designee will be given the opportunity to participate. Participants or their legal surrogates will be given the consent/assent during the screening visit. They will be given the opportunity to review the consent/assent and ask questions about clarification on any aspects of the study. Any additional questions they have will also be answered by the investigator prior to signing the consent/assent. Once the consent/assent form is signed, a signed and dated copy of the authorization form will be provided to the subject and another copy will be placed in the participant's medical record at the Affiliated Hospital of Xuzhou Medical University.

E. Randomization and Blinding

Based on the computer-generated random number table, participants are centrally randomized 1:1 to receive either active-tDCS group or sham-tDCS group. The allocation information is concealed in opaque envelopes and revealed until the investigators administer intervention to patients on the day before the surgery. Outcome assessors, the researchers who process data, other health care personnel, and patients are blinded to the treatment

allocation.

F. Sample Size Calculation

All analyses are conducted with the intention-to-treat principle. Our preliminary data showed that the incidence of perioperative anxiety was 51.4%, and we assumed that a 40% reduction in the incidence of perioperative anxiety after two stimulation of tDCS would be considered a response to treatment. Using a study power of 0.80 and a significance level of 0.05, we then derived that 88 patients per group were required by using PASS 15.0. Considering a 10% loss of follow-up, we aimed to recruit 98 participants in each group.

G. Statistical Analysis

Data analysis will perform in September 2023. The Kolmogorov-Smirnov test is used to assess normality, independent sample t-test is used if normally distributed data, and Mann-Whitney U test is used otherwise for non-normally distributed data. Continuous variables with normal distribution are represented by mean and standard deviation (SD), and variables with non-normal distribution are represented by median and interquartile range (IQR). Categorical variables are expressed as frequencies and proportions and analyzed using chi-square tests or Fisher exact tests.

Primary outcome is compared between the two groups using chi-square test or Fisher exact test, with differences between groups expressed as relative risk (RR) and 95% confidence interval (CI). The linear mixed-effects model is used to compare the secondary outcomes, with baseline (T0) values as covariates, treatment, time, and the interaction of time and treatment as fixed effects, and the subject's random intercept as a random effect to account for differences in time. The treatment-by-time interaction term is tested first. If significant, between- and within-treatment differences are tested for each time

point, and analyses are adjusted for multiple comparisons using the Bonferroni test for differences in within-treatment anxiety scores from baseline (T0). Otherwise, the main effect of treatment was tested next, and no Bonferroni correction was made for assessing the treatment effect at each time point. The difference and 95% confidence interval (CI) between medians were calculated with Hodges-Lehmann estimator. The relative risk (RR) and 95% CI were used to describe the differences in dichotomous outcomes. Data are analyzed using SPSS 25.0 (IBM Corp, Armonk NY) and hypothesis tests are two-sided at a level of 0.05.

H. Intervention with tDCS

The apparatus (machine manufacturer: Jiangxi Huaheng Jingxing Medical Technology Co.; machine specification model: MBM-I) is used in this trial. The electrostimulation is delivered through two electrodes placed in saline-soaked sponges. The electrodes are fixed by a stretchy hat, with the anode over the left DLPFC.

On the day before and the day of surgery, patients in the active-tDCS group receive 2 mA tDCS with a 30-s ramp-up phase at the beginning and a 30-s ramp-down phase at the end for 20 min. While patients in the sham-tDCS group only receive a 30-s ramp-up phase at the beginning and a 30-s ramp-down phase at the end of each session without a constant current of 2 mA for 20 min.

I. Anesthesia protocol

Standard monitoring procedures are initiated as soon as patients arrive in the operating room, including electrocardiography, invasive arterial blood pressure, pulse oximetry, and central venous catheterization.

The patients are administered general anesthesia with 0.5 μg/kg sufentanil, 0.3 mg/kg etomidate, and 1 mg/kg rocuronium, the tracheal

catheter is inserted after the bispectral index value decreased to less than 60, and the anesthesia machine is used for mechanical ventilation with the end-expiratory carbon dioxide partial pressure maintained between 35 and 45 mmHg. Patients undergo ultrasound-guided bilateral transversus abdominis plane block with 40 ml of 0.375% ropivacaine and are transferred to the PACU after surgery. Intravenous infusion of propofol at a rate of 4-6 mg/kg/h and remifentanil at a rate of 0.1-0.3 μg/kg/min, along with continuous inhalation of 1% sevoflurane are used to maintain bispectral index values between 40 and 60 for the anesthesia maintenance. If required, vasoactive drugs are used to maintain hemodynamic stability during anesthesia.

Postoperative treatment: The tracheal catheter is removed after the patients regain consciousness from anesthesia, ensuring an optimal tidal volume. Postoperative analgesia is achieved with a patient-controlled intravenous infusion of 1.5 μg/kg sufentanil and 6 mg tropisetron with saline to 100 mL. The background infusion rate is 2 mL/h and the self-controlled analgesic dose is 0.5 ml.

J. Outcome measures

The primary outcome is the incidence of perioperative anxiety in patients from the day of surgery to 3 days after surgery after two sessions of tDCS intervention. Perioperative anxiety was measured using the Hospital Anxiety and Depression Scale-Anxiety (HADS-A) **(Appendix 1)**. A HADS-A score of 8 or more is considered to be a patient experiencing anxiety. On the day before surgery, the researcher assesses the patient for anxiety in the ward treatment room (T0), followed by a second assessment of anxiety (T1) after the first tDCS intervention. On the day of surgery, the researcher conducted separate assessments before (T2) and after (T3) administering the second tDCS stimulus. Anxiety scores were assessed at two hours after surgery (T4), on the

first day after surgery (T5), the second day after surgery (T6), the third day after surgery (T7), and the third month after surgery (T8).

Secondary outcomes included anxiety scores (T1-T8), the incidence of postoperative delirium (POD, assessed by the Confusion Assessment Method [CAM] or Confusion Assessment Method intensive care unit [CAM-ICU] **[Appendix 2]** on the day of surgery and three days after surgery [T4-T7]); pain scores (assessed by the Numeric Rating Scale [NRS] **[Appendix 3]** at T4-T7); frail scores (assessed by the Fatigue, Resistance, Ambulation, Illness and Loss of Weight Index [FRAIL] **[Appendix 4]** at T5-T8); and sleep quality score (assessed by the Pittsburgh Sleep Quality Index [PSQI] **[Appendix 5]** at T4-T8).

K. Surgical Method

Laparoscopic colorectal tumor resection.

IV. Adverse Events

An adverse event (AE) is an unforeseen medical event that occurs to a patient or a clinical study subject. The tDCS has been established as a safe and tolerable form of non-invasive brain stimulation. Adverse events commonly consist of paraesthesia, such as mild tingling, burning and itching, as well as headaches, and transient skin redness. In this trial, the investigator observes the patient's facial expression as the patients begin to receive the stimulation (avoided indicative questions such as asking if there is tingling, discomfort, etc.).

V. DATE AND SAFETY MONITORING

Clinical research will formulate a corresponding data safety monitoring plan according to the size of the risk. In the implementation stage of clinical research, it will be to record all adverse events in detail, handle and track them

properly until they are properly resolved or the condition is stable, and report serious adverse events and unexpected events to the ethics committee, competent authorities and drug regulatory authorities in a timely manner as required. The principal investigator will periodically conduct a comprehensive review of all adverse events, and if necessary, an investigator meeting convened to assess the risks and benefits of the study. This experiment is a double-blind trial, and the blinding will be promptly unmasked if necessary to ensure the safety and rights of the subjects. We will arrange independent data monitors to monitor the research data, and high-risk studies will establish an independent data safety monitoring committee to monitor the accumulated safety data and efficacy data in order to determine whether to continue the study.

VI. FUNDING

This study was supported in part by grants from the National Key R&D Program of China (2021ZD0203100 to J-LC; 2022ZD0206200 to SZ); National Natural Science Foundation of China (NSFC81720108013, NSFC31771161, and NSFC81230025 to J-LC; NSFC82171227 and NSFC81300957 to HL; NSFC81771453 and NSFC31970937 to HXZ; NSFC82371536 to SZ); Jiangsu Provincial Special Program of Medical Science (BL2014029 to J-LC); Basic and Clinical Research Center in Anesthesiology of Jiangsu Provincial "Science and Education for Health" Project (J-LC); Zhejiang Provincial Natural Science Foundation (LY22H090019 to HL); Jiangsu Provincial Natural Science Foundation (BK20190047 to HXZ); the Priority Academic Program Development of Jiangsu Higher Education Institutions (19KJA610005 to HXZ); Distinguished Professor Program of Jiangsu (HXZ); Jiangsu Province Innovative and Entrepreneurial Talent Program and Jiangsu Province Innovative and Entrepreneurial Team Program (HXZ); Xuzhou Medical University start-up grant for excellent scientist (D2018010 and D2019025D to HXZ); the Natural Science Foundation of Shanghai (21ZR1411300 to YH); and

Shenkang Clinical Study Foundation of Shanghai (SHDC2020CR4061 to YH).

VII. INFORMATION CONFIDENTIALITY

Medical records will be kept in the hospital, and the investigators and ethics committees will be allowed to access the patient's medical records. Any public reporting of the results of this study will not disclose the patient's personal identity.

VIII. LITERATURE CITED

[1]American Psychological Association. Anxiety. Available at: http://www.apa.org/ topics/anxiety/. Accessed February 25, 2016

[2]E. Neeman, S. Ben-Eliyahu, Surgery and stress promote cancer metastasis: new outlooks on perioperative mediating mechanisms and immune involvement, Brain. Behav. Immun. 30 Suppl(Suppl) (2013) S32-40.

[3] X.R. Li, W.H. Zhang, J.P. Williams, T. Li, J.H. Yuan, Y. Du, J.D. Liu, Z. Wu, Z.Y. Xiao, R. Zhang, G.K. Liu, G.R. Zheng, D.Y. Zhang, H. Ma, Q.L. Guo, J.X. An, A multicenter survey of perioperative anxiety in China: Pre- and postoperative associations, J. Psychosom. Res. 147 (2021) 110528.

[4] A. Baumert, N. Buchholz, A. Zinkernagel, P. Clarke, C. MacLeod, R. Osinsky, M. Schmitt, Causal underpinnings of working memory and Stroop interference control: Testing the effects of anodal and cathodal tDCS over the left DLPFC, Cogn. Affect. Behav. Neurosci. 20(1) (2020) 34-48.

[5] S.J. Banks, K.T. Eddy, M. Angstadt, P.J. Nathan, K.L. Phan, Amygdala-frontal connectivity during emotion regulation, Soc. Cogn. Affect. Neurosci. 2(4) (2007) 303-12.

[6] N.M. Maldonado, P.J. Espejo, I.D. Martijena, V.A. Molina, Activation of ERK2 in basolateral amygdala underlies the promoting influence of stress on fear memory and anxiety: influence of midazolam pretreatment, Eur. Neuropsychopharmacol. 24(2) (2014) 262-70.

[7] J.P. Lefaucheur, A. Antal, S.S. Ayache, D.H. Benninger, J. Brunelin, F. Cogiamanian, M. Cotelli, D. De Ridder, R. Ferrucci, B. Langguth, P. Marangolo, V. Mylius, M.A. Nitsche, F. Padberg, U. Palm, E. Poulet, A. Priori, S. Rossi, M. Schecklmann, S. Vanneste, U. Ziemann, L. Garcia-Larrea, W. Paulus, Evidence-based guidelines on the therapeutic use of transcranial direct current stimulation (tDCS), Clin. Neurophysiol. 128(1) (2017) 56-92.

[8] G. D'Urso, A. Mantovani, S. Patti, E. Toscano, A. de Bartolomeis, Transcranial Direct Current Stimulation in Obsessive-Compulsive Disorder, Posttraumatic Stress Disorder, and Anxiety Disorders, J. ECT 34(3) (2018) 172-181.

Appendix 1. The Hospital Anxiety and Depression Scale

The Hospital Anxiety and Depression Scale

Name: Date:

Doctors are aware that emotions play an important part in most illnesses. If your doctor knows about these feelings he will be able to help you more.

This questionnaire is designed to help your doctor to know how you feel. Ignore the numbers printed on the left of the questionnaire. Read each item and underline the reply that comes closest to how you have been feeling in the past week.

Don't take too long over your replies: your immediate reaction to each item will probably be more accurate than a long thought-out response.

questions	answers	score
01. I feel tense or 'wound up' (A):	Most of the time	3
	A lot of the time	2
	From time to time, occasionally	1
	Not at all	0
02. I still enjoy the things I used to enjoy (D):	Definitely as much	0
	Not quite so much	1
	Only a little	2
	Hardly at all	3
03. I get a sort of frightened feeling as if something awful is about to happen (A):	Very definitely and quite badly	3
	Yes, but not too badly	2
	A little, but it doesn't worry me	1
	Not at all	0
04. I can laugh and see the funny side of things (D):	As much as I always could	0
	Not quite so much now	1
	Definitely not so much now	2
	Not at all	3
05. Worrying thoughts go through my mind (A):	A great deal of the time	3
	A lot of the time	2
	From time to time but not too often	1
	Only occasionally	0
06. I feel cheerful (D):	Not at all	3
	Not often	2
	Sometimes	1
	Most of the time	0

07. I can sit at ease and feel relaxed (A):	Definitely	0
	Usually	1
	Not often	2
	Not at all	3
08. 1 feel as if I am slowed down (D):	Nearly all the time	3
	Very often	2
	Sometimes	1
	Not at all	0
09. I get a sort of frightened feeling like 'butterflies' in the stomach (A):	Not at all	3
	Occasionally	2
	Quite often	1
	Very often	0
10. I have lost interest in my appearance (D):	Definitely	0
	I don't take so much care as I should	1
	I may not take quite as much care	2
	I take just as much care as ever	3
11. I feel restless as if I have to be on the move (A):	Very much indeed	3
	Quite a lot	2
	Not very much	1
	Not at all	0
12. I look forward with enjoyment to things (D):	As much as ever I did	3
	Rather less than I used to	2
	Definitely less than I used to	1
	Hardly at all	0
13. I get sudden feelings of panic (A):	Very often indeed	0
	Quite often	1
	Not very often	2
	Not at all	3
14. I can enjoy a good book or radio or TV programme (D):	Often	0
	Sometimes	1
	Not often	2
	Very seldom	3

"(A)" scores were used to screen for the presence of anxiety symptoms;

A: ______________

8~10: mild anxiety, 11~14: moderate anxiety, 15~21: severe anxiety

Appendix 2. The Confusion Assessment Method Instrument (CAM)

The Confusion Assessment Method Instrument

Acute onset

1. Is there evidence of an acute change in mental status from the patient's baseline?

Inattention*

2. A. Did the patient have difficulty focusing attention, for example, being easily distractible, or having difficulty keeping track of what was being said?

   Not present at any time during interview.

   Present at some time during interview, but in mild form.

   Present at some time during interview, in marked form.

   Uncertain.

   B. (If present or abnormal) Did this behavior fluctuate during the interview, that is, tend to come and go or increase and decrease in severity?

   Yes.

   No.

   Uncertain

   Not applicable.

   C. (If present or abnormal) Please describe this behavior:

Disorganized thinking

3. Was the patient's thinking disorganized or incoherent, such as rambling or irrelevant conversation, unclear or illogical flow of ideas, or unpredictable switching from subject to subject?

Altered level of consciousness

4. Overall, how would you rate this patient's level of consciousness?

   Alert (normal).

   Vigilant (hyper-alert, overly sensitive to environmental stimuli, startled very easily).

   Lethargic (drowsy, easily aroused).

   Stupor (difficult to arouse).

Coma (unarousable).

Uncertain.

Disorientation

5. Was the patient disoriented at any time during the interview, such as thinking that he or she was somewhere other than the hospital, using the wrong bed, or misjudging the time of day?

Memory impairment

6. Did the patient demonstrate any memory problems during the interview, such as inability to remember events in the hospital or difficulty remembering instructions?

Perceptual disturbances

7. Did the patient have any evidence of perceptual disturbances, for example, hallucinations, illusions, or misinterpretations (such as thinking something was moving when it was not)?

Psychomotor agitation

8. Part 1.

At any time during the interview, did the patient have an unusually increased level of motor activity, such as restlessness, picking at bedclothes, tapping fingers, or making frequent sudden changes of position?

Psychomotor retardation

8. Part 2.

At any time during the interview, did the patient have an unusually decreased level of motor activity, such as sluggishness, staring into space, staying in one position for a long time, or moving very slowly?

Altered sleep-wake cycle

9. Did the patient have evidence of disturbance of the sleep-wake cycle, such as excessive daytime sleepiness with insomnia at night?

* The questions listed under this topic were repeated for each topic where applicable.

Appendix 3. The Numeric Rating Scale (NRS)

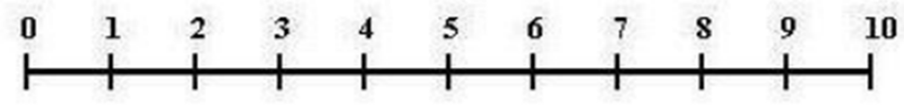

Appendix 4. The Fatigue, Resistance, Ambulation, Illness and Loss of Weight Index (FRAIL)

FRAIL SCALE

Fatigue	Are you fatigued?	All/most of the time = 1 Some/a little/none of the time = 0
Resistance	Do you have difficulty walking up one flight of stairs without assistance?	Yes = 1/No = 0
Ambulatory	Do you have difficulty walking 1 block without assistance?	Yes = 1/No = 0
Illness	Do you have more than 5 illnesses (confirm with doctor or medical records)?	Yes = 1/No = 0
Loss of Weight	Have you lost more than 5% of your weight in the past year?	Yes = 1/No = 0

Scoring: robust (score = 0), prefrail (score = 1–2), and frail (score = 3–5)

Appendix 5. Pittsburgh Sleep Quality Index (PSQI)

Pittsburgh Sleep Quality Index

Name： Date：

Instructions:

The following questions relate to your usual sleep habits during the past month only. Your answers should indicate the most accurate reply for the majority of days and nights in the past month.

Please answer all questions.

1. During the past month, when have you usually gone to bed at night?

USUAL BEDTIME：

2. During the past month, how long (in minutes) has it usually taken you to fall asleep each night?

NUMBER OF MINUTES ：

3. During the past month, when have you usually gotten up in the morning?

USUAL GETTING UP TIME ：

4. During the past month, how many hours of actual sleep did you get at night? (This may be different than the number of hours you spend in bed.)

HOURS OF SLEEP PER NIGHT ：

For each of the remaining questions, check the best response. Please answer all questions.

5. During the past month, how often have you had trouble sleeping because you...

(a) Cannot get to sleep within 30 minutes

Not during past month The Less than Once a week

Once or twice a week Three or more times a week

(b) Wake up in the middle of the night or early morning

Not during past month The Less than Once a week

Once or twice a week Three or more times a week

(c) Have to get up to use the bathroom

Not during past month The Less than Once a week

Once or twice a week Three or more times a week

(d) Cannot breathe comfortably

Not during past month The Less than Once a week

Once or twice a week Three or more times a week

(e) Cough or snore loudly

Not during past month The Less than Once a week

Once or twice a week Three or more times a week

(f) Feel too cold

Not during past month The Less than Once a week

Once or twice a week Three or more times a week

(g) Feel too hot

Not during past month The Less than Once a week

Once or twice a week Three or more times a week

(h) Had bad dreams

Not during past month The Less than Once a week

Once or twice a week Three or more times a week

(i) Have pain

Not during past month The Less than Once a week

Once or twice a week Three or more times a week

(j) Other reason(s), please describe:

How often during the past month have you had trouble sleeping because of this?

Not during past month The Less than Once a week

Once or twice a week Three or more times a week

6. During the past month, how would you rate your sleep quality overall?

Very good Fairly good Fairly bad Very bad

7. During the past month, how often have you taken medicine (prescribed or "over the counter") to help you sleep?

Not during past month The Less than Once a week

Once or twice a week Three or more times a week

8. During the past month, how often have you had trouble staying awake while driving, eating meals, or engaging in social activity?

Not during past month The Less than Once a week

Once or twice a week Three or more times a week

9. During the past month, how much of a problem has it been for you to keep up enough enthusiasm to get things done?

No problem at all Only a very slight problem

Only a very slight problem A very big problem

10. Do you have a bed partner or roommate?

No bed partner or roommate Partner/roommate in other room

Partner in same room, but not same bed Partner in same bed

If you have a roommate or bed partner, ask him/her how often in the past month you have had...

(a) Loud snoring

Not during past month The Less than Once a week

Once or twice a week Three or more times a week

(b) Long pauses between breaths while asleep

Not during past month The Less than Once a week

Once or twice a week Three or more times a week

(c) Legs twitching or jerking while you sleep

Not during past month The Less than Once a week

Once or twice a week Three or more times a week

(d) Episodes of disorientation or confusion during sleep

Not during past month The Less than Once a week

Once or twice a week Three or more times a week

(e) Other restlessness while you sleep: please describe

Not during past month The Less than Once a week

Once or twice a week Three or more times a week
