# Supplementary material for: Transcranial Direct Current Stimulation for Anxiety During Laparoscopic Colorectal Cancer Surgery: A Randomized Clinical Trial
Source: JAMA Netw Open. 2024 Apr 18;7(4):e246589. doi: 10.1001/jamanetworkopen.2024.6589 (PMC12527477; doi:10.1001/jamanetworkopen.2024.6589)
Supplement: Supplement 2. — eTable 1. Comparison of Hospital Anxiety and Depression Scale-Anxiety Subscale (HADS-A) Scores of Patients With Moderate to Severe Anxiety at T0 Between Groups eTable 2. Binary Logistic Regression Analysis to Identify Postoperative Anxiety at T4 eTable 3. Adverse Events in the Trial eFigure 1. Timeline of the Trial and Apparatus Used in This Test eFigure 2. Comparison of Mean Scores of Anxiety, Numeric Rating Scale, Pittsburgh Sleep Quality Index (PSQI), and Frailty Index of Patients Between the Two Groups eFigure 3. Forest Plot of the Subgroup Analysis [file jamanetwopen-e246589-s002.pdf]

Supplemental Online Content

Li C, Tao M, Chen D, et al. Transcranial direct current stimulation for anxiety during laparoscopic colorectal cancer surgery. *JAMA Netw Open.* 2024;7(4):e246589. doi:10.1001/jamanetworkopen.2024.6589

eTable 1. Comparison of Hospital Anxiety and Depression Scale-Anxiety Subscale (HADS-A) Scores of Patients With Moderate to Severe Anxiety at T0 Between Groups

eTable 2. Binary Logistic Regression Analysis to Identify Postoperative Anxiety at T4

eTable 3. Adverse Events in the Trial

eFigure 1. Timeline of the Trial and Apparatus Used in This Test

eFigure 2. Comparison of Mean Scores of Anxiety, Numeric Rating Scale, Pittsburgh Sleep Quality Index (PSQI), and Frailty Index of Patients Between the Two Groups

eFigure 3. Forest Plot of the Subgroup Analysis

This supplemental material has been provided by the authors to give readers additional information about their work.

eTable 1. Comparison of Hospital Anxiety and Depression Scale-Anxiety Subscale (HADS-A) Scores of Patients With Moderate to Severe Anxiety at T0 Between Groups

Variable	Active-tDCS (n = 38)	Sham-tDCS (n = 36)	*P* value[a]
**HADS-A scores, mean (SD)[de]**			
**T0**	13.1 (1.5)	13.5 (2.1)	.35
**T1**	11.9 (1.5)[b]	13.5 (2.1)	<.001
**T2**	11.3 (2.0)[b]	15.4 (2.2)	<.001
**T3**	10.2 (1.9)[b]	15.4 (2.2)	<.001
**T4**	9.1 (1.7)[b]	13.0 (2.5)	<.001
**T5**	8.5 (1.7)[b]	12.1 (2.2)[c]	<.001
**T6**	7.8 (1.6)[b]	10.5 (2.1)[c]	<.001
**T7**	7.1 (1.4)[b]	9.2 (2.0)[c]	<.001
**T8**	6.7 (1.2)[b]	8.5 (1.9)[c]	<.001

Abbreviation: HADS-A, Hospital Anxiety and Depression Scale-Anxiety subscale (range: 0-21, with higher scores indicating more anxiety); tDCS, transcranial direct current stimulation; T0, before the first tDCS intervention (active or sham) on the day before the operation; T1, after the first tDCS intervention on the day before the operation; T2, before the second tDCS intervention on the day of the operation; T3, after the second tDCS intervention on the day of the operation; T4, 2 hours after the operation; T5, the first day after the operation; T6, the second day after the operation; T7, the third day after the operation; T8, the third month after the operation.

[a]Active tDCS group vs sham tDCS group at each time point.

[b]Refers to P <.001, when each time point vs. T0 within the active tDCS group.

[c]Refers to P <.001, when each time point vs. T0 within the sham tDCS group.

[d]HADS-A scores of patients who were moderate to severe anxiety at T0 were compared in linear mixed-effects models between groups (P for group < .001, P for time < .001, P for interaction < .001).

[e]The anxiety degree is as follows: 8-10 score is mild anxiety, 11-14 score is moderate anxiety and 15-21 score is severe anxiety.

eTable 2. Binary Logistic Regression Analysis to Identify Postoperative Anxiety at T4

Variable	Univariable analysis		Multivariable analysis	
	Unadjusted OR (95% CI)	*P* value	Adjusted OR (95% CI)	*P* value
**Group**	0.30 (0.17-0.15)	<.001	0.05 (0.01-0.22)	<.001
**Preoperative anxiety**	28.85 (13.27-62.70)	<.001	149.22 (28.52-780.69)	<.001
**Age**	1.02 (0.58-1.79)	.94	NA	NA
**Sex**	1.81 (1.01-3.25)	.05	1.68 (0.62-4.56)	.31
**Hemoglobin**[b]	1.01 (0.53-1.92)	.97	NA	NA
**Tumor metastasis**	0.86 (0.47-1.56)	.62	NA	NA
**Ileostomy**	1.98 (1.09-3.59)	.03	3.22 (1.20-9.23)	.02
**ICU admission**	2.11 (0.49-9.08)	.37	NA	NA
**Adverse event**	2.09 (1.17-3.73)	.01	1.25 (0.47-3.36)	.65
**Remedial analgesia**	2.97 (1.58-5.58)	.001	1.72 (0.58-5.14)	.33
**PSQI**	16.61 (3.77-73.14)	<.001	5.77 (0.85-39.17)	.07
**FRAIL**	1.65 (0.93-2.91)	.09	3.79 (1.33-10.82)	.01
**History of Surgery**	0.98 (0.54-1.79)	.99	NA	NA
**ASA classification**	1.97 (0.77-5.07)	.16	NA	NA
**Education level**	2.10 (1.06-4.15)	.03	1.65 (0.50-5.41)	.41

Abbreviation: NA, not applicable (the multivariable section in the model only used variables with *P* value in univariable analysis <.10); OR, odds ratio; CI, confidence interval; ICU, Intensive Care Unit; PSQI, Pittsburgh Sleep Quality Index; FRAIL, Fatigue, Resistance, Ambulation, Illness and Loss of Weight Index; ASA, American Society of Anesthesiologists physical status classification; T4, two hours after surgery.

[a] Specific subgroups: age (≤65 vs. >65), sex (male vs. female), hemoglobin (non-anemia vs. anemia), tumor metastasis (yes vs. no), ileostomy (yes vs. no), ICU admission (yes vs. no), adverse event (yes vs. no), remedial analgesia (yes vs. no), PSQI (≤15 vs. >15), FRAIL (robust vs. prefrail or frail), history of surgery (yes vs. no), ASA score (II vs. III), education level (≤ 9 vs. > 9).

[b] Normal hemoglobin values are 120-160 g/L for adult men and 110-150 g/L for adult women.

eTable 3. Adverse Events in the Trial

Characteristic	Active-tDCS (n = 98)	Sham-tDCS (n = 98)	*P* value
**Nausea and vomiting**	9 (9.2)	15 (15.3)	.28
**Dizziness**	0 (0.0)	1 (1.0)	1.00
**Fever**	20 (20.4)	20 (20.4)	1.00
**Hypoxia**	0 (0.0)	1 (1.0)	1.00
**Wound infection**	2 (2.0)	1 (1.0)	1.00
**Postoperative pelvic abscess**	2 (2.0)	1 (1.0)	1.00
**Intracranial hemorrhage**	0 (0.0)	1 (1.0)	1.00
**All-cause 3-month mortality**	2 (2.0)	3 (3.1)	1.00
**Skin irritation at the stimulation site**	11 (11.2)	3 (3.1)	.03

Abbreviation: tDCS, transcranial direct current stimulation.

eFigure 1. Timeline of the Trial and Apparatus Used in This Test

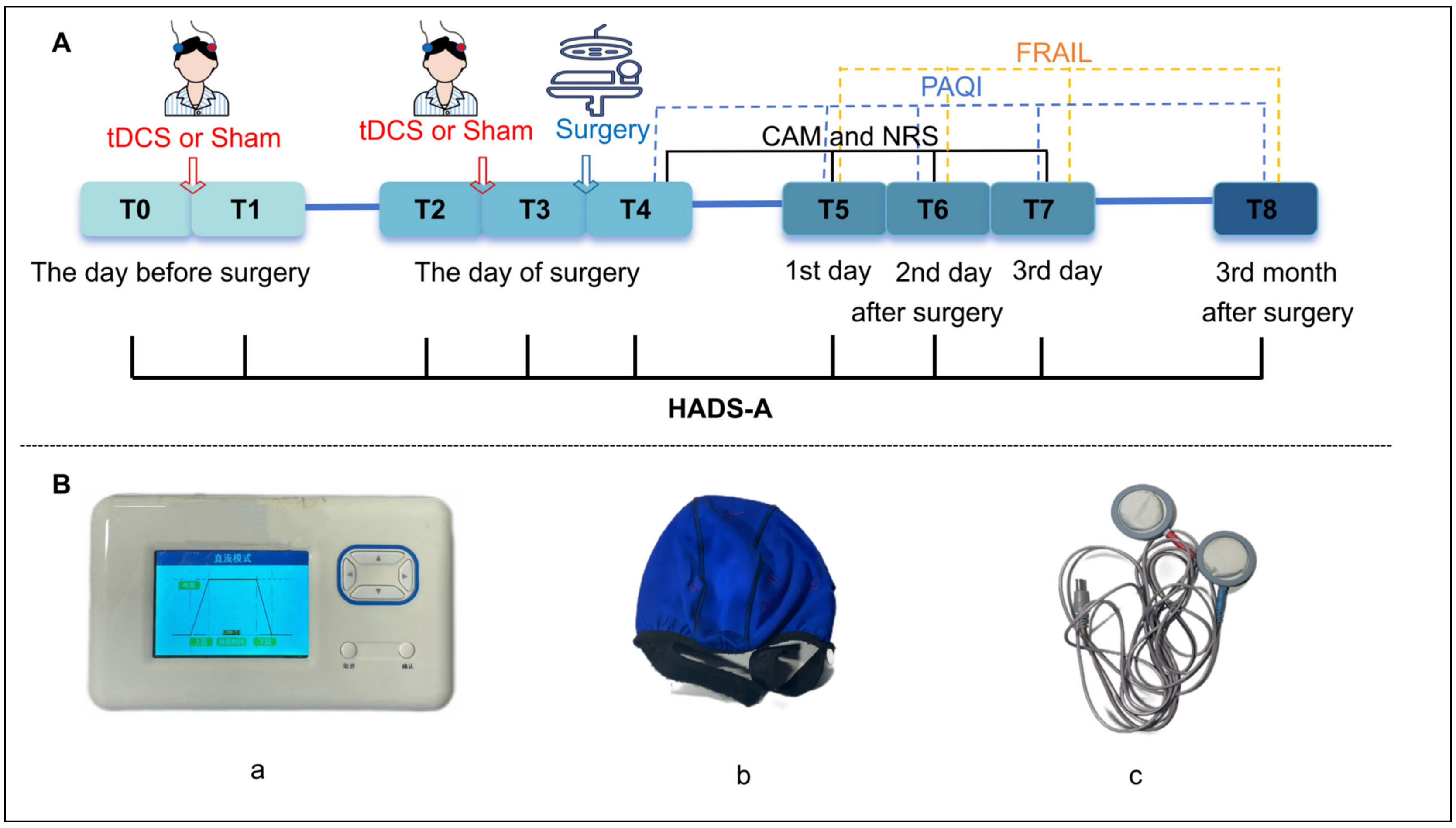

A, the experimental design and timeline. B, the apparatus used in this test. a, the mainframe of the apparatus; b: stretchy hat used to fix the electrodes; c, electrodes.

Abbreviation: CAM, Confusion Assessment Method; FRAIL, Fatigue, Resistance, Ambulation, Illness and Loss of Weight Index (ranging from 0 [robust] to 5 [worst frail]); HADS-A, Hospital Anxiety and Depression Scale-Anxiety subscale (range: 0-21, with higher scores indicating more anxiety); NRS, Numeric Rating Scale (ranging from 0 [no pain] to 10 [worst pain]); PSQI, Pittsburgh Sleep Quality Index (ranging from 0 to 21, with the higher the score, the worse the sleep quality); tDCS, transcranial direct current stimulation; T0, before the first tDCS intervention (active or sham) on the day before the operation; T1, after the first tDCS intervention on the day before the operation; T2, before the second tDCS intervention on the day of the operation; T3, after the second tDCS intervention on the day of the operation; T4, 2 hours after the operation; T5, the first day after the operation; T6, the second day after the operation; T7, the third day after the operation; T8, the third month after the operation.

eFigure 2. Comparison of Mean Scores of Anxiety, Numeric Rating Scale, Pittsburgh Sleep Quality Index (PSQI), and Frailty Index of Patients Between the Two Groups

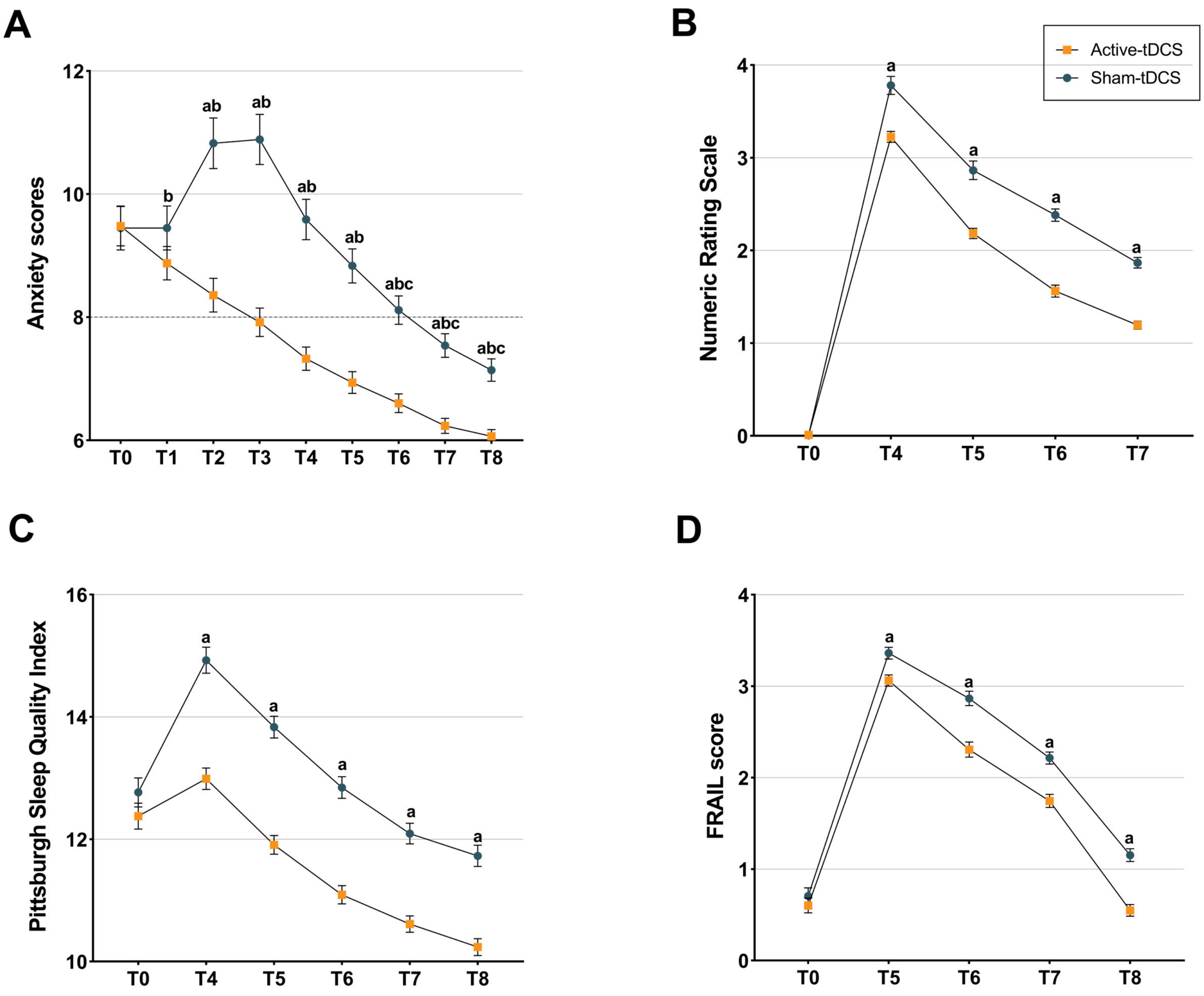

Error bars represent the SE of the mean.

Abbreviation: FRAIL, Fatigue, Resistance, Ambulation, Illness and Loss of Weight Index (ranging from 0 [robust] to 5 [worst frail]); NRS, Numeric Rating Scale (ranging from 0 [no pain] to 10 [worst pain]); PSQI, Pittsburgh Sleep Quality Index (ranging from 0 to 21, with the higher the score, the worse the sleep quality); tDCS, transcranial direct current stimulation; T0, before the first tDCS intervention (active or sham) on the day before the operation; T1, after the first tDCS intervention on the day before the operation; T2, before the second tDCS intervention on the day of the operation; T3, after the second tDCS intervention on the day of the operation; T4, 2 hours after the operation; T5, the first day after the operation; T6, the second day after the operation; T7, the third day after the operation; T8, the third month after the operation.

aRefers to P <.01, when active tDCS group vs. Sham tDCS group at each time point.

bRefers to P <.001, when each time point vs. T0 within the active tDCS group.

cRefers to P <.001, when each time point vs. T0 within within the sham tDCS group.

eFigure 3. Forest Plot of the Subgroup Analysis

Subgroup	Active-tDCS (Events/Total)	Sham-tDCS (Events/Total)	Relative Risk (95% CI)	*P* interaction
**Sex**				**.31**
Male	17/60	31/64	0.56 (0.34, 0.94)	
Female	13/38	25/34	0.47 (0.29, 0.76)	
**PSQI**				**.07**
≤15	21/89	45/84	0.44 (0.29 0.67)	
>15	9/9	11/14	1.27 (0.97, 1.67)	
**Ileostomy**				**.02**
Yes	9/29	17/33	0.60 (0.32, 1.34)	
No	21/69	39/65	0.51 (0.34, 0.76)	
**Adverse event**				**.65**
Yes	15/38	24/40	0.66 (0.41, 1.05)	
No	15/60	32/58	0.45 (0.28, 0.74)	
**Remedial analgesia**				**.33**
Yes	12/25	25/35	0.67 (0.43, 1.06)	
No	18/73	31/63	0.50 (0.31, 0.80)	
**FRAIL**				**.01**
Robust	17/60	26/50	0.55 (0.34, 0.88)	
Prefrail or Frail	13/38	30/48	0.55 (0.34, 0.90)	

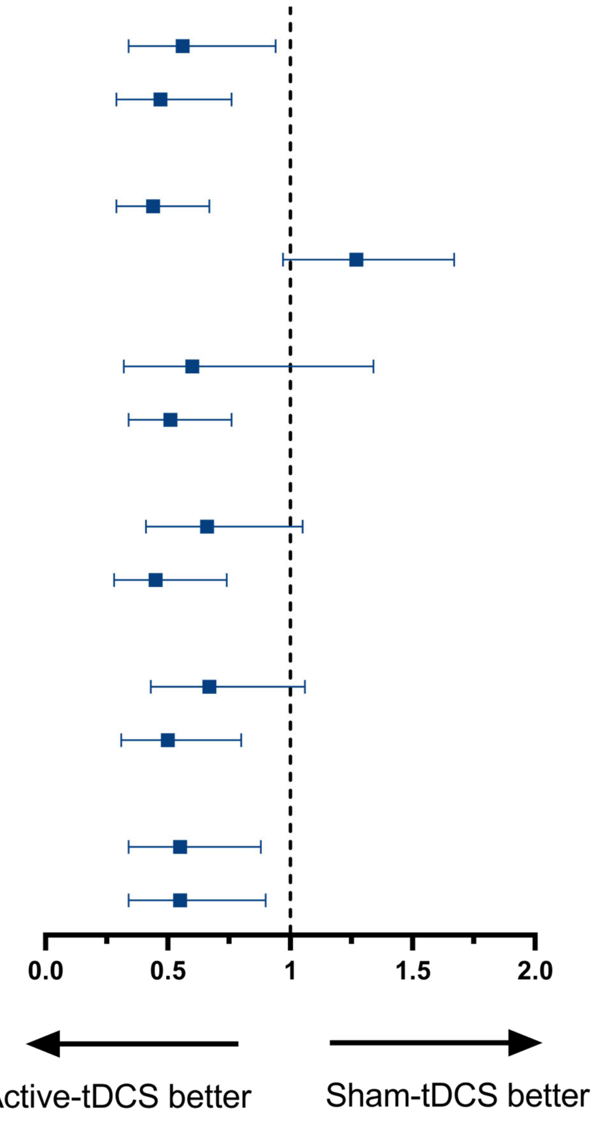

Binary logistic regression analysis shows the heterogeneity of tDCS in that particular subgroup of postoperative anxiety at T4 (2 hours after the operation), and the effect of the intervention method (relative risk [95% CI]) is presented separately in each subgroup. The interaction term is a test of whether the effect of the experimental intervention is statistically different in significance between subgroups.

Abbreviation: FRAIL, Fatigue, Resistance, Ambulation, Illness and Loss of Weight Index (ranging from 0 [robust] to 5 [worst frail]); PSQI, Pittsburgh Sleep Quality Index (ranging from 0 to 21, with the higher the score, the worse the sleep quality); tDCS, transcranial direct current stimulation; T4, 2 hours after the operation.
