# Supplementary material for: Transcranial Direct Current Stimulation for Anxiety During Laparoscopic Colorectal Cancer Surgery: A Randomized Clinical Trial
Source: JAMA Netw Open. 2024 Apr 18;7(4):e246589. doi: 10.1001/jamanetworkopen.2024.6589 (PMC12527477; doi:10.1001/jamanetworkopen.2024.6589)
Supplement: Supplement 3. — Data Sharing Statement [file jamanetwopen-e246589-s003.pdf]

Data Sharing Statement

Li. Transcranial Direct Current Stimulation for Anxiety During Laparoscopic Colorectal Cancer Surgery. *JAMA Netw Open*. Published April 18, 2024.
doi:10.1001/jamanetworkopen.2024.6589

Data
Data available: No
